# Successful Use of Chimeric Antigen Receptor T-cell (CAR-T) Therapy With Lisocabtagene Maraleucel in a Renal Transplant Recipient With Refractory/Relapsed Diffuse Large B-Cell Lymphoma (DLBCL)

**DOI:** 10.7759/cureus.103667

**Published:** 2026-02-15

**Authors:** Waqqas Tai, Fatima Chaudhry, Nesreen Shahrour, Jessica Thomas, Afoma Anyadibe, Anuoluwa Oyetoran, Swathi Gopishetty, Precious Idogun, Dilip Samarapungavan, Jassim H Sarmad, Ishmael Jaiyesimi

**Affiliations:** 1 Hematology and Oncology, Corewell Health William Beaumont University Hospital, Royal Oak, USA; 2 Oncology, Eastern Virginia Medical School, Norfolk, USA; 3 Oncology, Georgetown University School of Medicine, Washington D.C., USA; 4 Hematology and Oncology, Corewell Health, Royal Oak, USA; 5 Nephrology, Corewell Health William Beaumont University Hospital, Royal Oak, USA; 6 Pathology, Corewell Health William Beaumont University Hospital, Royal Oak, USA

**Keywords:** car-t cells in hematological malignancies, diffuse large b cell lymphoma (dlbcl), kidney transplant, liso-cel, lymphoma, post-transplant lymphoproliferative disorders

## Abstract

This case describes a 53-year-old male with end-stage renal disease who developed monomorphic post-transplant lymphoproliferative disorder (PTLD) in the form of diffuse large B-cell lymphoma (DLBCL) after kidney transplantation. Despite initial treatment with rituximab, cyclophosphamide, doxorubicin, vincristine, prednisone (R-CHOP), the patient’s disease progressed, and he was referred for chimeric antigen receptor (CAR) T-cell therapy with lisocabtagene maraleucel (liso-cel). Given his post-transplant status, his immunosuppressive agents (tacrolimus and mycophenolate) were held, and prednisone was tapered to 5 mg daily to maintain minimal baseline immunosuppression. After lymphodepleting chemotherapy and liso-cel infusion, the patient experienced no significant toxicities, including cytokine release syndrome (CRS) or immune effector cell-associated neurotoxicity syndrome (ICANS). This case underscores the potential of CAR-T therapy for refractory/relapsed DLBCL in post-transplant patients, emphasizing the need for careful immunosuppressive management to balance graft protection and treatment efficacy.

## Introduction

Diffuse Large B-Cell Lymphoma (DLBCL) is the most common subtype of non-Hodgkin lymphoma and is characterized by aggressive clinical behavior [1]. The standard first-line chemoimmunotherapy regimen is rituximab, cyclophosphamide, doxorubicin, vincristine, and prednisone (R-CHOP). This regimen induces remission in approximately 50-60% of patients; however, about 30-40% experience relapsed or refractory (R/R) disease [2]. R/R DLBCL is associated with significant morbidity and mortality, particularly among those with primary refractory disease or early relapse, who face a poor prognosis. Over the past decade, CD19-directed chimeric antigen receptor T-cell (CAR-T) therapy has become an established treatment for R/R DLBCL, inducing durable remissions in a subset of heavily pretreated patients and leading to FDA approval of axicabtagene ciloleucel (axi-cel), tisagenlecleucel (tisa-cel), andlisocabtagene maraleucel (liso-cel) after at least two prior lines of therapy [3-7]. Despite these advancements, management of R/R DLBCL in solid organ transplant recipients represents a distinct clinical challenge, as therapeutic decision making must account for baseline immunosuppression, heightened infection risk, and the potential for allograft rejection.

## Case presentation

This is a 53-year-old African American male with a significant medical history of type 2 diabetes mellitus, hypertension, and end-stage renal disease (ESRD) secondary to diabetic nephropathy. He initially required hemodialysis from 2010 to 2015 and subsequently underwent a deceased donor kidney transplant in June 2015. His post-transplant course was complicated by chronic allograft nephropathy, leading to progressive renal dysfunction.

In April 2024, during evaluation for a second renal transplant, a routine computed tomography (CT) scan of the abdomen and pelvis revealed extensive peritoneal and mesenteric nodularity, along with abdominal and pelvic lymphadenopathy. An ultrasound-guided omental biopsy confirmed a diagnosis of monomorphic post-transplant lymphoproliferative disorder (PTLD), specifically diffuse large B-cell lymphoma (DLBCL) with a high proliferative index (80-90%), Epstein-Barr virus (EBV)-negative status, and a double-expressor phenotype (*BCL2/MYC* co-expression). Fluorescent in situ hybridization (FISH) analysis showed *MYC* gain in 37.5% of nuclei without rearrangement, ruling out double-hit lymphoma. Staging positron emission tomography-computed tomography (PET-CT) in May 2024 demonstrated high-grade, bulky extranodal disease involving the omentum, mesentery, small bowel, as well as widespread nodal involvement in the neck, chest, abdomen, and pelvis. Routine labs at the time of diagnosis were significant for mild normocytic anemia and chronic kidney disease (CKD) (Table 1).

**Table 1:** Complete blood count and comprehensive metabolic panel at time of diagnosis April 2024 eGFR CKD-EPI: Estimated glomerular filtration rate

Laboratory parameter	Patient Value	Units	Reference Range
Complete blood count			
White blood cell count	3.8	×10⁹/L	3.5–10.1
Red blood cell count	4.2	×10¹²/L	4.31–5.48
Hemoglobin	11	g/dL	13.5–17.0
Hematocrit	36.2	%	40.1–50.1
Mean corpuscular volume	86	fL	80–100
Mean corpuscular hemoglobin	26	pg	28–33
Mean corpuscular hemoglobin concentration	30	g/dL	32–36
Red cell distribution width (RDW-CV)	16	%	12–15
Platelet count	202	×10⁹/L	150–400
Nucleated red blood cells	0	%	≤0.0
Comprehensive metabolic panel			
Sodium	135	mmol/L	135–145
Potassium	5.1	mmol/L	3.5–5.2
Chloride	106	mmol/L	98–111
Carbon dioxide (CO₂)	19	mmol/L	20–29
Anion gap	10	mmol/L	5–17
Glucose	104	mg/dL	70–99 (fasting)
Blood urea nitrogen	67	mg/dL	7–25
Creatinine	3.83	mg/dL	0.60–1.30
eGFR CKD-EP	18	mL/min/1.73 m²	≥60
Calcium	10.1	mg/dL	8.5–10.5
Total protein	7	g/dL	6.4–8.3
Albumin	4	g/dL	3.5–5.1
Globulin	3	g/dL	2.2–4.0
Albumin/globulin ratio	1.3	ratio	1.0–2.5
Alkaline phosphatase	90	U/L	33–120
Aspartate aminotransferase (AST)	17	U/L	<35
Alanine aminotransferase (ALT)	16	U/L	9–47
Total bilirubin	0.3	mg/dL	0.3–1.2

**Figure 1:**
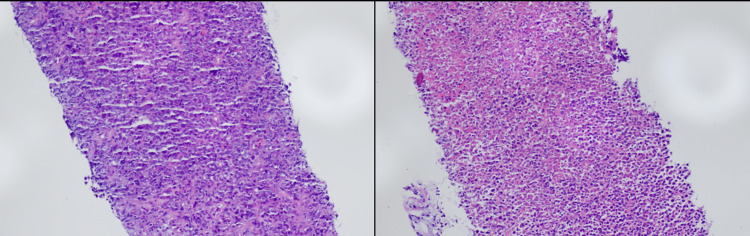
Retroperitoneal soft tissue biopsy Diffuse large B cell lymphoma (DLBCL) germinal center B-cell type with necrosis, Epstein-Barr virus (EBV) negative, post-transplant setting (monomorphic posttransplant lymphoproliferative disorder). Hematoxylin and Eosin (H&E) (20X) images showing diffuse sheets of large cells with foci of geographic necrosis.

**Figure 2:**
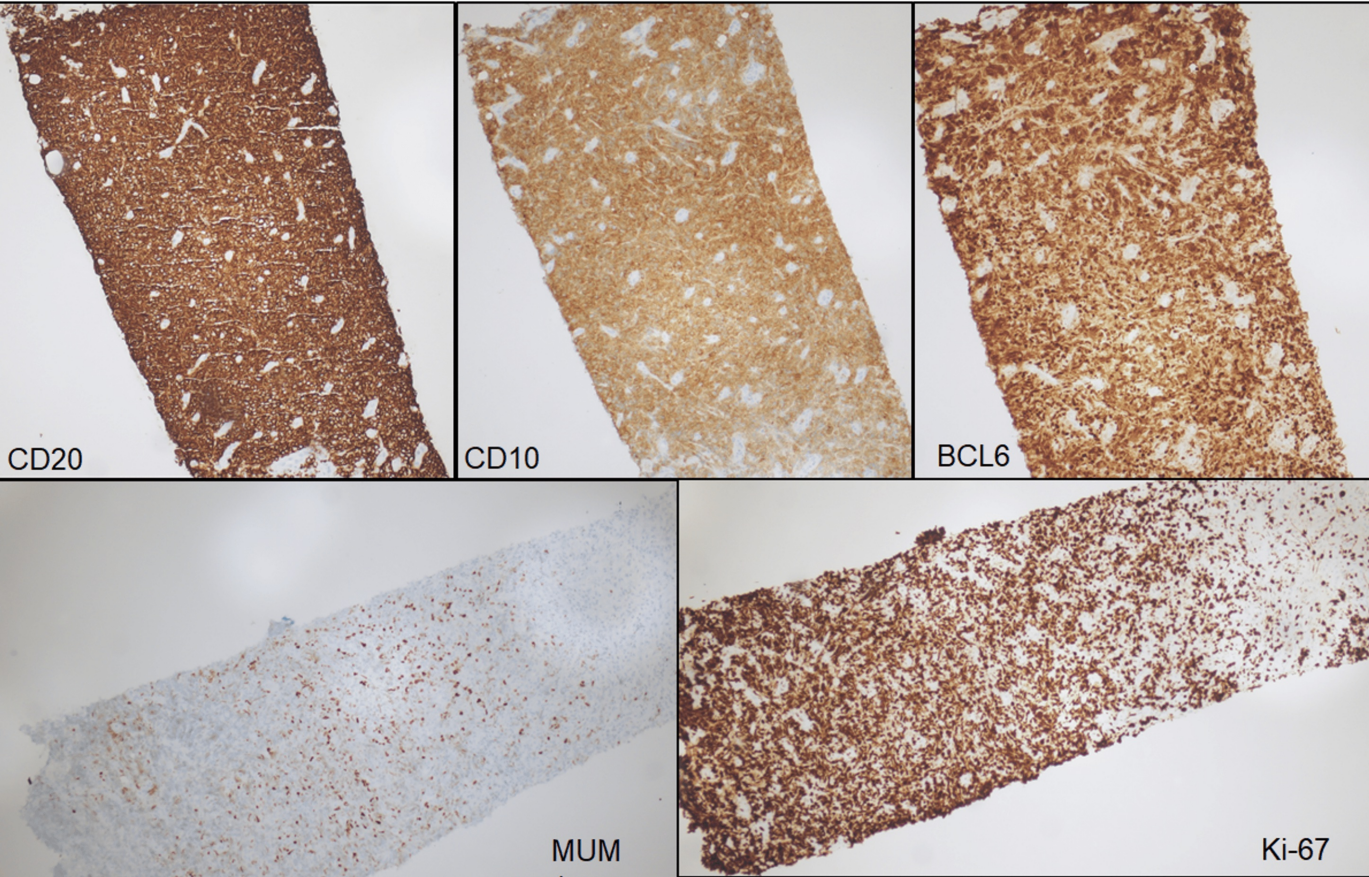
Retroperitoneal soft tissue biopsy The large lymphoma cells are positive for CD20 (10X), CD10 (10X), BCL6 (10X) consistent with germinal center B cell type by Hans algorithm. MUM1 (10X) is expressed in less than 30% of cells and deemed predominantly negative. Ki-67 (10X) proliferation index is high at approximately 80-90% within viable cells.

**Figure 3:**
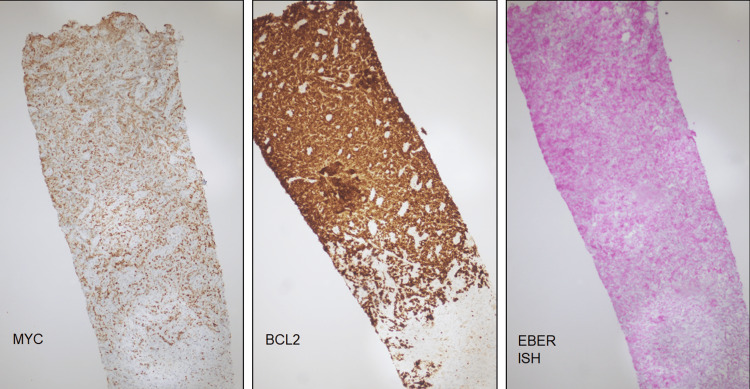
Retroperitoneal soft tissue biopsy MYC (10X) is variably overexpressed in approximately 40-50% of cells and BCL2 is diffusely positive in keeping with a double expressor profile. In situ hybridization for Epstein Barr Virus encoded mRNA or EBER ISH was negative (10X). All controls worked appropriately. EBER-ISH: Epstein-Barr virus early RNA in-situ hybridization

The patient was promptly started on systemic therapy with R-CHOP. Concurrently, his immunosuppression (tacrolimus and mycophenolate) was held, and he was maintained on prednisone 10 mg. An interim PET-CT after cycle five showed a favorable partial metabolic response but with residual disease (Deauville score 5) [8]. He completed six cycles of R-CHOP by the end of August 2024. However, a follow-up PET scan in November 2024 revealed disease progression with new lesions in the liver, retroperitoneum, pelvis, musculature, and seminal vesicle. The brain MRI was negative. A CT-guided biopsy of a retroperitoneal lymph node confirmed recurrent DLBCL, germinal center B-cell type, EBV-negative, with necrosis, consistent with a post-transplant setting (Figures 1-3).

**Figure 4:**
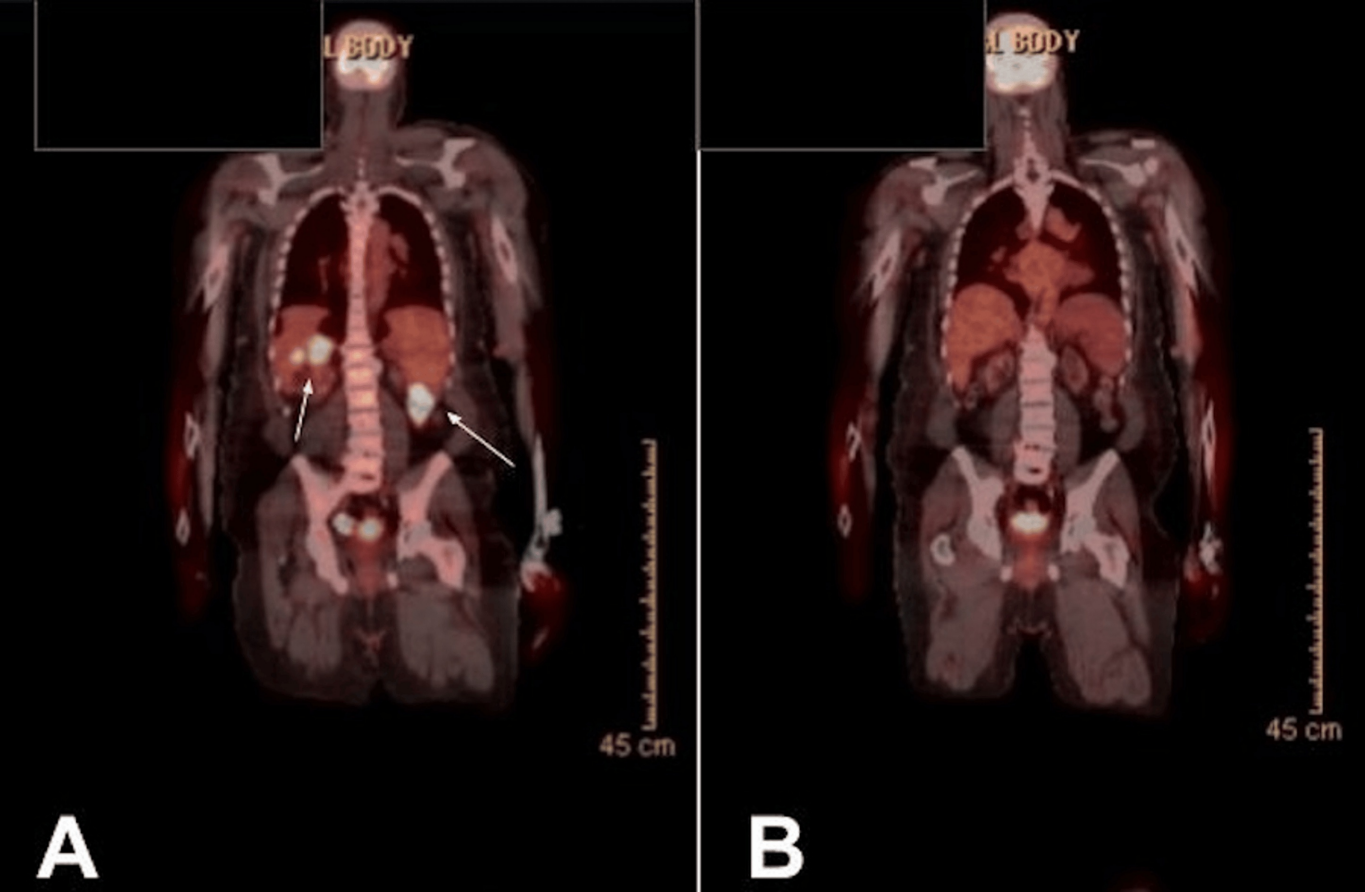
PET scan A) Pre–CAR-T (January 2025): widespread hypermetabolic nodal and extranodal disease involving the abdomen, retroperitoneum, pelvis, and liver, consistent with progressive diffuse large B-cell lymphoma. (B) Post–CAR-T at day 100 (May 2025): complete metabolic response, with resolution of all previously identified hypermetabolic lesions. PET: positron emission tomography

Given the disease progression on first-line therapy, the patient was referred for chimeric antigen receptor T-cell (CAR-T) therapy evaluation, specifically for liso-cel. Pre-CAR-T cardiac evaluation revealed a reduced ejection fraction (EF) of 25%, delaying treatment initiation. He was managed with guideline-directed medical therapy by cardiology, with his EF improving to 35% by January 2025. Following multidisciplinary discussions involving cardiology and transplant nephrology, the patient elected to proceed with liso-cel. During this time, he received bridging therapy with polatuzumab and rituximab. A third PET scan in January 2025 demonstrated continued disease progression (Figure 4) [3].

In February 2025, the patient was admitted for inpatient monitoring, received lymphodepleting chemotherapy, and subsequently underwent liso-cel infusion. He was closely monitored by the transplant nephrology team. Although the CAR-T protocol was steroid-free, prednisone was tapered to 5 mg daily to maintain minimal baseline immunosuppression and protect the renal allograft.

The inpatient course was uneventful. The patient experienced no ICANS or CRS. He was safely discharged on day 7 post-infusion. He continues to do well, with stable renal function, and day 100 PET-CT showing complete metabolic response [3] (Figure 4). 

## Discussion

Emerging clinical trial data support the use of CD19 directed CAR T-cell therapy earlier in the treatment course of R/R DLBCL, challenging the historical reliance on salvage chemoimmunotherapy and autologous transplantation. More recently, the TRANSFORM trial demonstrated that second line liso-cel significantly improved event-free survival and complete response rates (74% vs. 43%) compared with salvage chemoimmunotherapy followed by autologous stem cell transplantation in patients with primary refractory or early relapsed DLBCL [9]. A single-arm phase 2 PILOT study further showed high response rates with liso-cel in patients with R/R DLBCL who were ineligible for high-dose chemotherapy and transplantation due to comorbidities or frailty, supporting its use in medically complex populations [10].

DLBCL treatment in solid organ transplant recipients requires careful balancing of therapeutic efficacy with transplant safety. Chronic immunosuppression contributes to the development of PTLD by impairing immune surveillance. Yet, it is essential to prevent allograft rejection. Notably, monomorphic EBV-negative PTLD, as seen in this patient, is less responsive to immunosuppression reduction alone and typically requires systemic multi-agent chemotherapy, even under reduced immunosuppression [11].

Managing such cases requires close collaboration between the oncology and transplant teams to taper immunosuppressive agents sufficiently to restore anti-lymphoma immune activity while minimizing the risk of rejection. Active immunosuppressive drugs can impair T-cell function and proliferation, potentially reducing the efficacy of CAR-T cell therapy. In a multicenter retrospective analysis of relapsed PTLD patients treated with CAR-T cell therapy, 64% had their immunosuppression completely discontinued prior to CAR-T cell infusion [12]. While this approach enhances CAR-T cell activity, it also increases the risk of graft rejection. Indeed, three of 22 patients in the study experienced allograft rejection following treatment [12]. However, these episodes were manageable, and some patients were able to resume reduced immunosuppression after achieving lymphoma control. These findings suggest that, with appropriate patient selection, careful monitoring, and multidisciplinary management, CAR T-cell therapy is both feasible and effective for PTLD even in the setting of potential allograft rejection.

Given the patient’s R/R DLBCL following standard therapy, CAR-T cell therapy was pursued due to the lack of alternative treatment options, as the disease was refractory to R-CHOP. Allogeneic stem cell transplantation was considered too high-risk in the context of an immunosuppressed solid organ transplant recipient. Liso-cel was selected as the CAR-T cell product for several reasons. It has demonstrated significant efficacy in DLBCL, achieving an overall response rate of 73% and acomplete response rate of 53%, with most patients having chemotherapy-refractory disease [8]. These high remission rates underscore liso-cel’s potent anti-lymphoma activity, which was necessary in this case to effectively target CD19-expressing malignant B cells.

Alternative CAR-T cell products presented limitations. Axi-cel, although effective, is associated with higher rates of severe cytokine release syndrome (CRS) and neurologic toxicity, likely due to its CD28 costimulatory domain [13]. Tisa-cel, while associated with a more favorable safety profile, has shown lower efficacy in refractory DLBCL. The incidence of grade ≥3 CRS in clinical trials was 2% with liso-cel, compared to 13% with axi-cel and 22% with tisa-cel [8]. Therefore, liso-cel was chosen to optimize therapeutic efficacy while minimizing the risk of severe toxicities.

Krishnamoorthy et al. reported three solid organ transplant recipients (pancreas, kidney, and heart) with refractory PTLD treated with axi-cel CAR-T cells, all of whom developed severe CRS refractory to standard management with dexamethasone and Tocilizumab therapy [14]. Each patient developed acute kidney injury (AKI), two required renal replacement therapy, none achieved a response, and all died from treatment-related toxicities [14]. Melilli et al. similarly described a kidney transplant recipient who developed AKI after tisa-cel. Biopsy showed mononuclear infiltrates without CD19+ CAR T cells, suggesting graft injury from a systemic inflammatory response rather than direct CAR-T-mediated toxicity. Renal function recovered with steroids, but PTLD relapsed, and the patient died seven months later [15].

To the best of our knowledge, the first reported use of liso-cel in a renal transplant recipient with R/R DLBCL was described by Portuguese et al. [16]. A 47-year-old man with three prior kidney transplants and stage IV EBV-negative DLBCL PTLD received R-CHOP (partial response), rituximab and the chemotherapy drugs ifosfamide, carboplatin, and etoposide phosphate (R-ICE) for progression, and bridging pola-bendamustine-rituximab before lymphodepletion with cyclophosphamide/fludarabine and liso-cel infusion. He was maintained on tacrolimus, mycophenolate mofetil, and prednisone, with mycophenolate mofetil discontinued at PTLD diagnosis and tacrolimus held before leukapheresis. After CAR-T, low-dose prednisone was continued, and tacrolimus was later reintroduced at reduced exposure [16]. After CAR-T infusion, prednisone was continued at 5 mg/day, tacrolimus was restarted on day 65 with a lower target trough level, and immunosuppression was further reduced during relapse management. He achieved a complete response by three months. However, relapse occurred at eight months and was treated with reduced immunosuppression and localized radiation. A new obturator muscle lesion was discovered 8 months postoperatively, despite confirmation of peripheral CAR-T activity. This finding supports the notion that immunosuppression may diminish the efficacy of CAR-T therapy and prevent sustained remission versus other mechanisms that lead to refractory disease. [16]. 

Various studies have explored strategies to balance immunosuppression with CAR-T cell therapy, yielding mixed results. Mamlouk et al. described a case of a kidney transplant recipient with DLBCL whose immunosuppressive therapy was discontinued prior to CAR-T cell administration [17]. The patient achieved complete remission, and kidney function remained stable off immunosuppressants. However, by week 21, serum creatinine levels had increased. Further investigation revealed cell-mediated rejection of the allograft, followed by antibody-mediated rejection seven weeks later. In contrast, Hernani et al. and Guy et al. both reported successful outcomes in kidney transplant recipients with treatment-refractory DLBCL after withdrawal of immunosuppression [18,19]. In the case reported by Hernani et al., the patient achieved complete remission one month after CAR-T cell therapy, with stable kidney function at 10-month follow-up [18]. In the case by Guy et al., complete remission was attained by day 120. Tacrolimus was reinitiated 10 days later, and kidney function remained preserved, with the patient maintaining remission 20 months post-treatment [19].

Luttwak et al. reported on two kidney transplant recipients and one liver transplant recipient who received tacrolimus concurrently with CAR-T cell therapy [20]. All three patients maintained satisfactory levels of CD19-directed CAR-T cells despite ongoing treatment with tacrolimus. Two patients achieved complete remission following the initial infusion, while one experienced a partial response. This report demonstrates the feasibility of simultaneous immunosuppression and CAR-T cell therapy. However, the durability of the response remains uncertain.

Lastly, Dang et al. successfully administered an immunosuppressive regimen alongside CAR-T cell therapy in a heart transplant recipient with PTLD [21]. The patient had no disease relapse at six months post-treatment; however, her course was complicated by persistent pancytopenia, requiring hematopoietic cell transplantation (HCT) and close follow-up.

## Conclusions

No standardized approach currently exists for balancing immunosuppression with CAR-T cell therapy. Success and complications have been observed with strategies involving withdrawal, reinitiation, or continued use of immunosuppressants. Ultimately, optimal management requires multidisciplinary collaboration, vigilant monitoring, and ongoing reassessment of both graft function and disease status. Long-term studies are needed to evaluate the durability of remission and graft viability under various immunosuppressive regimens.
